# *Aedes albopictus *and the reemergence of Dengue

**DOI:** 10.1186/1471-2458-12-72

**Published:** 2012-01-24

**Authors:** Giovanni Rezza

**Affiliations:** 1 Department of Infectious Diseases, Istituto Superiore di Sanità, Roma, Italy

## Abstract

Dengue is a vector-borne disease that is estimated to affect millions of individuals each year in tropical and subtropical areas, and it is reemerging in areas that have been disease-free for relatively long periods of time. In this issue of the journal, Peng et al. report on a Dengue outbreak in a city in southern China that had been disease-free for more than two decades. The infection, which was due to serotype 1, was introduced by a traveler from South-east Asia and transmitted by *Aedes albopictus*, the Asian tiger mosquito. Compared to *Aedes aegypti*, which is the most important vector of Dengue, *Ae albopictus *is a less competent vector of arboviruses, and the epidemics it causes are milder. However, *Ae albopictus *is becoming an increasingly important vector because of its rapidly changing global distribution. In particular, the worldwide trade in second hand tires, which often contain water and are an ideal place for eggs and larvae, has been a key factor in the large-scale conquest of *Ae albopictus*, which easily adapts to new environments, even in a temperate climate. This expansion is creating new opportunities for viruses to circulate in new areas, becoming a common cause of epidemics in *Ae aegypti*-free countries, from Hawaii to Mauritius. The outbreak in China, like similar events, was mild and short-lived. Because epidemics due to *Ae albopictus *are milder, the replacement of *Ae aegypti *with the tiger mosquito could even result in public-health benefits. However, there is no solid evidence of this, and the milder course of the outbreak could be in part explained by the relatively short duration of the hot season in some affected areas. Since it is almost impossible to prevent *Ae albopictus *from being introduced in a country, mosquito-control measures at local level remain the most effective means of controlling arbovirus outbreaks.

## Commentary

Dengue is an emerging infectious disease that is estimated to affect 50-100 million individuals each year in tropical and subtropical areas [1]. Demographic and societal changes, such as population growth, urbanization, and modern transportation, have greatly contributed to the increased incidence and geographical spread of Dengue virus infection in recent decades.

In this issue of "BMC Public Health", H.J. Peng et al. [2] report on the reemergence of Dengue in a city in southern China that had been disease-free for more than two decades. The infection, which had been introduced by a traveler from South-east Asia, was transmitted by *Aedes albopictus*, the Asian tiger mosquito.

*Ae albopictus *is generally believed to be a less efficient vector of arboviruses than *Ae aegypti*, the most important vector of Dengue. In particular, it is considered an inefficient vector of Dengue because it is not as well adapted to urban domestic environments and is less anthropophilic than *Ae aegypti*. In fact, it usually feeds on a single individual, and that it also feeds on animals decreases the probability of its feeding on humans [3]. By contrast, *Ae aegypti *females are highly anthropophilic and often feed on several persons before obtaining enough blood to complete a gonotrophic cycle. This tendency toward multiple feeding may contribute to the explosive nature of Dengue outbreaks in areas where *Ae aegypti *is present [4,5], whereas outbreaks in places where only *Ae albopictus *is present tend to be mild. This has led to the hypothesis that the spread of *Ae albopictus *and the consequent replacement of *Ae aegypti *populations in many places could actually result in benefits for public health. However, there is no solid evidence of this. Moreover, since *Ae albopictus *adapts better than *Ae aegypti *to temperate climates, outbreaks caused by the Asian tiger mosquito may occur also in areas where *Ae aegypti *is not present and where infection control is favoured by the arrival of cold weather. This could explain in part why outbreaks due to *Ae albopictus *are usually smaller than those due to *Ae aegypti*. Finally, it should be mentioned that specific *Ae albopictus *populations may sometimes develop a high vector capacity, as suggested by a massive outbreak of Dengue that was propagated by this mosquito on La Réunion in 1977 [6]. A mutation increasing the fitness in *Ae albopictus *was identified for another mosquito-borne virus, the Chikungunya virus, during an epidemic on La Réunion in 2005; a virus strain with the same mutation also caused an outbreak in north-eastern Italy [7,8].

Despite the fact that is considered to be a less efficient vector, *Ae albopictus *is becoming more important in causing Dengue outbreaks as a consequence of a rapid changes in its overall distribution. The Asian tiger mosquito hails from East and Southeast Asia, where it originally lived at the edges of forests, breeding in tree holes and other natural reservoirs. Centuries ago it spread to Madagascar and Indian Ocean islands, yet in the past 50 years it has spread to all inhabited continents, in large part as a result of increased global air travel and seaborne trade. Furthermore, a worldwide trade in second hand tires, which often contain water and are an ideal place for eggs and larvae, has been a key factor in the large-scale conquest of *Ae albopictus *[9]. The eggs are also resistant to drought and can survive until the tires reach their destination. In newly infested areas, the mosquito has adapted easily to human settlements, where pots, vases and buckets can act as breeding sites, provided that there is a bit of vegetation. Moreover, the eggs can also survive cold winters because they go into a state of dormancy or "diapause", allowing the *Ae albopictus *mosquito to persist in areas with a temperate climate.

In the last decade, *Ae albopictus *has been the vector in outbreaks in different areas of the world, from Hawaii [10] to Mauritius [11]. In China, an outbreak of Dengue transmitted by *Ae albopictus *was reported in the Ningbo area just a few years ago [12]. *Ae albopictus *was also the single vector in simultaneous outbreaks of DENV and CHIKV in Gabon and in Madagascar [13,14]. These recent Dengue outbreaks have had characteristics in common with the outbreak reported in this issue of the journal: they occurred in places that had been Dengue-free for a number of years; the virus was introduced by viremic travelers; and the epidemic course was mild and short-lived.

In Hawaii, where Dengue had been absent since the mid-1940s, an outbreak due to *Ae albopictus *occurred in 2001, and it was directly linked to a large DENV-1 epidemic on the Society Islands, French Polynesia, 4,400 km south of Hawaii. However, the outbreak in Hawaii was less severe than the other outbreaks caused by similar strains elsewhere in the Pacific, where *Ae aegypti *was the principal mosquito vector [10].

In Mauritius, DENV-2 reemerged in 2009, more than 10 years after the last outbreak had occurred in the 1970s. The reemergence was due to the introduction of DENV-1 by unrecognized infective travelers. *Ae albopictus*, which is widely distributed in Mauritius, was the probable vector, although the rapid increase in cases in June is more consistent with an *Ae aegypti*-borne outbreak. Nonetheless, the outbreak was short-lived as a result of control measures and the arrival of cooler and drier weather [11].

The 2004 outbreak of Dengue in Ningbo was the first outbreak since 1929. The virus was introduced by a person who had returned from Thailand, and a high density of mosquitoes contributed to the outbreak. The isolated strain differed from other DENV-1 strains from Guangdong and Hainan. As in the study by Peng [2], molecular techniques supported the epidemiological clues about the geographic origin of the virus. Mosquito density decreased due to eradication of larva infestation and cold weather. In May 2005, Dengue virus RNA could not be found in the pools of *Ae albopictus *collected from households and ponds around dwellings of Dengue cases. From November 2004 to November 2005, no new Dengue cases were reported, indicating the cessation of the outbreak [12].

The prevention and control of outbreaks caused by *Ae albopictus *is a challenge. Preventing mosquitoes from entering *Ae albopictus*-free countries is also important, though medium-distance travel in automobiles and trucks cannot be efficiently controlled and controlling long-distance infestation by limiting international tire trade would have important economic implications. Thus health promotion remains the first option, given that eliminating breeding sites, such as flowerpots and vases, is effective, as is spraying insecticides, whereas sterile insect technology is still under evaluation. In conclusion, the globalization of humans and mosquitoes is playing an important role in the reemergence of Dengue, and the expansion of the area of activity of the Asian tiger mosquito is creating new opportunities for viruses such as Dengue and Chikungunya to circulate in new areas.

## Pre-publication history

The pre-publication history for this paper can be accessed here:

http://www.biomedcentral.com/1471-2458/12/72/prepub
